# Host body mass, not sex, affects ectoparasite loads in yellow-necked mouse *Apodemus flavicollis*

**DOI:** 10.1007/s00436-023-07958-5

**Published:** 2023-09-13

**Authors:** Milena Zduniak, Sarah Serafini, Aleksandra Wróbel, Rafał Zwolak

**Affiliations:** 1 Department of Systematic Zoology, Adam Mickiewicz University in Poznań, Uniwersytetu Poznańskiego 6, 61-614 Poznań, Poland; 2 Department of Zoology, Poznań University of Life Sciences, Wojska Polskiego 71C, 60-625 Poznań, Poland

**Keywords:** Sex-biased parasitism, Ectoparasites, *Ixodes ricinus*, Siphonaptera, *Apodemus*, Small mammals

## Abstract

We investigated the presence and potential causes of sex bias in ectoparasite infestations in the yellow-necked mouse *Apodemus flavicollis*. We compared the natural tick and flea burdens of male and female mice in a temperate beech forest and assessed whether the observed differences were driven by host sex or body mass. We found that males were more heavily infested by ticks compared to female mice. However, this difference was driven by host body mass, and not sex itself. Host body mass positively correlated with flea loads, but there was no evidence of sex bias in flea abundance. In addition, the abundance of both ticks and fleas infesting yellow-necked mice changed over time, both seasonally (month to month) and annually (year to year). Our results underscore the importance of the sexual size dimorphism and the parasite taxon as the primary factors that influence the occurrence of sex-biased parasitism in small mammals.

**Supplementary Information:**

The online version contains supplementary material available at 10.1007/s00436-023-07958-5.

## Introduction

Sex-biased parasitism has been observed in numerous bird and mammal species (Zuk and McKean 1996; Poulin 1996; Schalk and Forbes 1997; Morand and Poulin 1998; Klein 2000; Wilson et al. 2002; Krasnov et al. 2012). However, the mechanisms behind this pattern remain a subject of debate, primarily because identifying the driving factor can be difficult if it is correlated with other unrelated traits (Zuur et al. 2010; Dormann et al. 2013). Moreover, parasite populations are affected by a number of factors, such as host population density, habitat selection, social or reproductive strategies, and behavioral types, that can interact with host gender (Klein 2004; Gutowsky et al. 2015; Wat et al. 2020). Consequently, unraveling the mechanisms behind sex-biased parasitism has proven to be a challenging task.

The sexual size dimorphism is among the factors that can affect parasite loads. Host body size and condition can impose constraints on the growth and composition of the parasite communities because they represent both the resource and the habitat for parasite populations (Brailsford and Mapes 1987; Price 1990; Christe et al 2003; Krasnov et al. 2005a, b; Bourgoin et al. 2021). As a consequence of intrasexual competition and the action of sex hormones, males are larger than females in most species of mammals (Weckerly 1998; Badyaev 2002; Isaac 2005). They are also frequently more parasitized than females (Schalk and Forbes 1997; Krasnov et al. 2012). Thus, it can be challenging to conclude whether parasites preferably infest males or simply choose larger individuals, who often happen to be males.

Another well-known explanation for sex-biased parasitism is the higher immunocompetence observed in females. This phenomenon is common among many vertebrate taxa (Zuk and McKean 1996; Poulin 1996; Waterman et al. 2013) and is associated with the action of sex hormones: estrogens stimulate immunity while androgens depress it (Folstad and Karter 1992; Schalk and Forbes 1997; Klein 2000). Steroid sex hormones may also affect resistance to diseases by altering the expression of major histocompatibility complex (MHC) genes (Klein 2000). Since immunity is a crucial defense mechanism against parasite infections, sexual hormones may indirectly affect the richness and abundance of parasite communities harbored by male and female hosts.

Finally, the life cycles and biology of parasites can also shape their interaction with the host. This relationship can also vary, depending on environmental conditions (Leung and Poulin 2008). For some parasites, it may be easier or more advantageous to inhabit males than females, depending on the sex-specific behavioral or physiological traits of the host. Additionally, as male and female hosts can interact differently with their environment, varying habitat qualities can also affect parasite transmission in a sex-specific manner. Therefore, it is essential to consider how parasite and habitat-specific traits mediate the interactions between male and female hosts and their parasites.

The purpose of this study was to compare the ectoparasite burden of male and female yellow-necked mice (*Apodemus flavicollis*) and to determine whether any potential gender bias is driven by the sex or body mass of the host. Our research is based on a similar project by Harrison et al. (2010), where authors estimated natural tick loads of wild wood mouse (*Apodemus sylvaticus*) populations in Irish mixed broadleaf and coniferous forest. Their results suggested that differences in parasite burdens between males and females were due to sex-related differences in body mass, not the sex itself. In this study, we follow their methodology and conduct analogical analyses to test if similar patterns occur in a congeneric rodent, *A. flavicollis,* in a temperate beech forest in Poland. As such, this study is a quasi-replication (Nakagawa and Parker 2015; Palmer 2000) of the research conducted by Harrison and colleagues (2010). Furthermore, we tested whether similar patterns are found in flea infestations of *A. flavicollis*. Our study species exhibits sexual size dimorphism (Schulte-Hostedde 2007), therefore we expected male-biased parasite burdens and predicted that both tick and flea numbers would be higher in males due to their greater body mass, not because of their sex. Our specific questions were as follows:

i. Do males carry higher ectoparasite loads than females?
ii. Does the sex bias in ectoparasite infestation persist after accounting for host sexual dimorphism?
iii. What is the relationship between male body mass and ectoparasite loads?
iv. What is the relationship between female body mass and ectoparasite loads?
v. Do the above relationships differ for ticks and fleas?

## Materials and methods

### Study site

This study took place in Forest Inspectorate Łopuchówko, Buczyna district, located in Greater Poland Voivodeship, N-W Poland. The maximum altitude at the study site is 143 m above sea level and the landscape is mostly flat or hilly. The temperatures range from an average of -2.5 °C in January to 18.2 °C in July, and the annual precipitation averages 520 mm. The study sites were situated in managed forests, primarily consisting of European beech (*Fagus sylvatica),* along with other species, such as pedunculate oak (*Quercus robur)*, red oak (*Quercus rubra)*, European hornbeam (*Carpinus betulus),* and sycamore maple (*Acer pseudoplatanus)* (categorized as habitat 9130, ‘Asperulo-Fagetum’ according to the EU Habitat Directive).

### Small mammal live-trapping and ectoparasite sampling

We established six trapping grids, each with 100 live traps, arranged in a 10 × 10 pattern, with 10-m spacing between the traps. To minimize the movement of mice between the grids, each grid was located at least 300 m apart. Trapping was carried out during three summer seasons (July–August 2018–2020). One trapping session consisted of four or five nights per site, and we conducted three (2018–19) or five (2020) trapping sessions per site. The total trapping effort amounted to 30,000 trapnights (9,000 in 2018 and 2019, and 12,000 in 2020).

At the first capture, all animals were assigned to species and marked with unique aluminum ear tags (National Band and Tag Company, mouse tags type 1005–1). We recorded the body mass of all individuals at each capture using the PESOLA scale (0.5 g accuracy), and visually determined their sex and reproductive status (scrotal or non-scrotal males, lactating, pregnant or nonpregnant females, and juveniles of both sexes). Shrews (*Sorex araneus* and *S. minutus*) were released unmarked.

After recording data on body mass and reproductive condition, we collected all fleas found on the host and in the handling bag. We then counted all ticks attached to the host, which were primarily located on the head and ears, though we searched the entire body. A random subset of 20 ticks was collected from each mouse to identify the tick species (fleas were not identified to species in this study) using laboratory molecular methods. Total genomic DNA was extracted from each tick individually using the ammonium hydroxide method (Rijpkema and Bruinink 1996). The tick species were determined using sequence data from the fragment of the cytochrome *c* oxidase subunit I (COI). The material was sequenced using Ion Torrent S5 System (Thermo Fisher, USA) and the results were compared with GenBank reference sequences.

### Statistical analysis

All statistical analyses were performed with R in RStudio IDE (R Core Team 2018; RStudio Team 2020). We used generalized linear mixed models (GLMMs, Bolker et al. 2009) implemented via the glmmTMB package (Brooks et al. 2017; Magnusson et al. 2017) and assessed fit with DHARMa and performance packages (Hartig and Hartig 2017; Lüdecke et al. 2021). To separate the influence of gender and body mass on ectoparasite burdens, we followed the statistical approach used by Harrison et al. (2010) with these adjustments:

- We fitted our models to both tick and flea data.
- We used the negative binomial error distribution with a log-link function.
- We included additive effects of month and year effects to control for seasonal and year-to-year changes in ectoparasite numbers (Langley and Fairley 1982; Gray 1991; Herrero-Cófreces et al. 2021).
- We adjusted the structure of the tick models to zero-inflated count data to account for the excess of zeros.
- To account for the nested data structure (Schielzeth and Nakagawa 2013), we included random effects of an individual mouse and trapping site.

Because pregnancy can confound the relationship between body mass, sex, and parasitism (Harrison et al. 2010), we excluded pregnant mice from the data set. We assessed pregnancy based on two traits: i) visibly enlarged belly, and ii) increased body mass compared to other trappings of the same individual. We also excluded juveniles from the data, we based our selection on body mass because we found pelage color to be overly subjective. We chose 15 g of body mass as a cut-off value between juveniles and adults (Pucek et al. 1993). However, the growth rate and the onset of reproduction in the yellow-necked mouse vary with food availability and other environmental factors (Gliwicz 1988; Balčiauskienė et al. 2009; Sawicka-Kapusta 1968; Ferrari et al. 2004), therefore, we explored the sensitivity of our results to different values of this threshold.

To address our first question (i. whether males carry higher ectoparasite loads), we fitted “model 1”, which tested the influence of host sex on tick load without considering the effect of host body mass. To address question two (ii. whether there is a sex bias in tick and flea loads after controlling for the effect of body mass), we paired males and females with equal weight. If an exact match was impossible, we paired individuals with a difference of no more than 0.5 g. No mouse was paired twice within one trapping session, but we allowed the same individual to be paired again in other sessions. We created analogical datasets for both ticks and fleas. We ran the paired model (“model 2”) using host sex as explanatory variable, with the pair ID as random effect. To tackle questions iii and iv – are heavier males/females more parasitized? – we divided the dataset into males and females and ran two models:”model 3″ to check the effect of body mass within the male sex, and “model 4″ to assess the effect of body mass within the female sex (the numeration of models follows Harrison et al. 2010). To address question v. we compared the effect sizes of the models fitted for tick and flea data with results obtained by Harrison et al. 2010 and other similar studies.

## Results

The small mammal community at our study sites was dominated by *Apodemus flavicollis* (66.9% of captured individuals) and bank vole (*Myodes glareolus*) (31.7% of individuals), followed by other species such as common vole (*Microtus arvalis*) *common shrew* (*Sorex araneus*), *Eurasian pygmy shrew* (*S. minutus*), and striped field mouse (*Apodemus agrarius*). In total, we captured 1873 unique mice, 200 in 2018 (116 males, 80 females, 4 unassessed), 880 in 2019 (414 males, 465 females, 1 unassessed), and 793 in 2020 (421 males, 370 females, 2 unassessed). The average body mass was higher in male compared to female yellow-necked mice (30.6 g vs. 26.0 g, t = -21.471, 95% CI: -5.07 – -4.22, df = 5732.9, P < 0.001).

### Effect of sex and body mass on tick loads

**Fig. 1:**
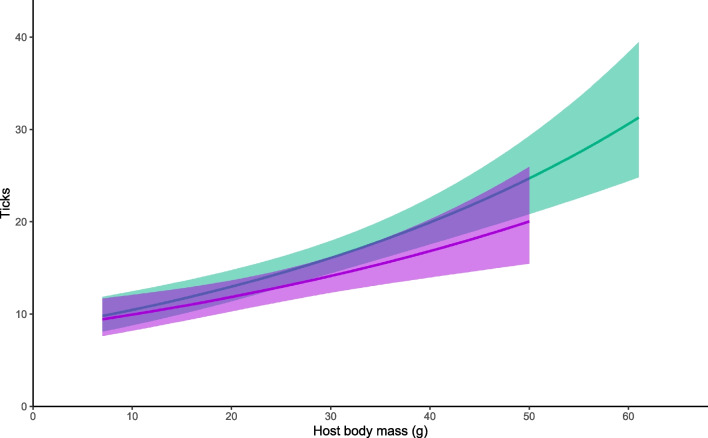
Estimated tick abundance in male (green) and female (violet) *A. flavicollis*. Shading corresponds to a 95% confidence interval. See Table 1, models 3 and 4, for more details

Overall (including recaptures), we sampled ticks 2079 times: 1065 times from male hosts and 1014 times from females. *Ixodes ricinus* was the only tick species found. According to model 1, that is without accounting for body mass, males carry more ticks on average than females (males 16.1 ticks, 95% CI: 14.3 – 18.1 vs. females: 12.9 ticks, 95% CI: 11.4 – 14.6; z = 4.95, P = 0.001). After controlling for the effect of body mass (model 2 that paired males and females of the same weight), the sex bias did not longer persist (z = 1.484, P = 0.138). The two models (3 and 4) in which we tested both sexes separately detected a positive association of body mass with tick loads in both males and females (males: z = 6.305, P < 0.001, N = 663; females: z = 3.757, P < 0.001, N = 561; Fig. 1). The effect of month and year was significant in every model (Supplementary Table 1, Supplementary Materials Fig. 1).

**Table 1:** Factors influencing the abundance of ticks infesting yellow-necked mice (*A. flavicollis*). Model (1) estimates the effect of sex without controlling for body mass, model (2) controls for body mass by pairing males with females of the same mass, model (3) estimates the effect of body mass on tick abundance in male hosts, and model (4) does the same for females. All models controlled the effect of month and year. Juveniles were excluded at the 15 g threshold. Random effects always included individual host and trapping site

Model	Effect	N	β ± SE	Ρ
1	Sex	1224	0.216 ± 0.044	< 0.001***
2	Sex (paired)	481	0.080 ± 0.054	0.138
3	Body mass	663 (only males)	0.021 ± 0.003	< 0.001***
4	Body mass	561 (only females)	0.017 ± 0.005	< 0.001***

*p < 0.05; **p < 0.01; ***p < 0.001

### Effect of sex and body mass on flea burdens

**Fig. 2:**
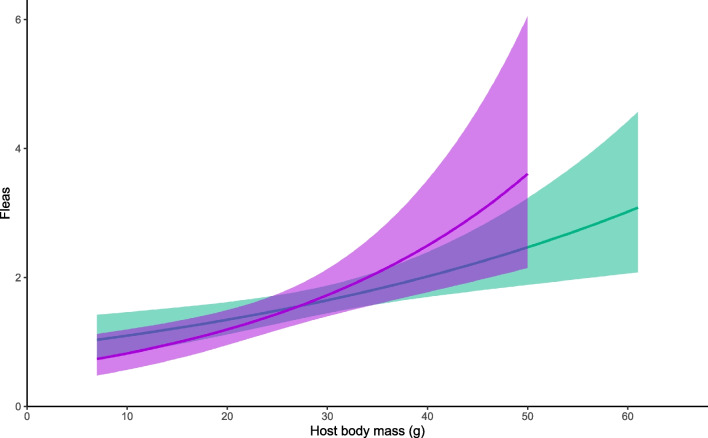
Estimated flea abundance in male (green) and female (violet) *A. flavicollis*. Shading corresponds to a 95% confidence interval. See Table 2 (models 3 and 4) for more details

Overall (including recaptures), we sampled fleas 2010 times: 1023 times from males and 987 times from females. In contrast to ticks, Model 1 did not reveal a sex bias in flea infestation (z = 0.97, P = 0.332). Similarly, Model 2 (with males and females paired by mass) did not find the effect of sex (z = -1.146, P = 0.271). However, when the sexes were tested separately (models 3 and 4), body mass was positively associated with both male (z = 3.230, p = 0.001) and female flea loads (z = 3.640, P < 0.001; see Fig. 2). We observed a significantly higher number of fleas in 2018, and flea abundance decreased in August compared to June and July (Supplementary Table 2, Supplementary Fig. 2).

**Table 2:** Factors influencing the abundance of fleas infesting yellow-necked mice (*Apodemus flavicollis).* Model (1) estimates the effect of sex without taking into account the body mass of the host, model (2) controls for body mass by pairing males with females of the same mass, model (3) estimates the effect of body mass on flea numbers harbored by male hosts, and model (4) does the same for females. All models controlled the effect of month and year. Juveniles were excluded at the 15 g threshold. Random effects always included individual host and trapping site

Model	Effect	N	β ± SE	Ρ
1	Sex	1224	0.082 ± 0.085	0.332
2	Sex (paired)	481	-0.146 ± 0.133	0.272
3	Body mass	663 (only males)	0.020 ± 0.006	0.001**
4	Body mass	561 (only females)	0.037 ± 0.010	< 0.001***

*p < 0.05; **p < 0.01; ***p < 0.001

## Discussion

We observed a male bias in tick loads of yellow-necked mice. However, when we accounted for differences in body mass, this pattern no longer persisted. This result indicates that sex-biased parasitism in this system is driven primarily by body mass, rather than other sex-related traits. In the case of flea abundance, we did not find any sex-related effects: both males and females carried similar flea loads, even when accounting for body mass. Only the host’s body mass had a significant impact on flea loads.

**Fig. 3:**
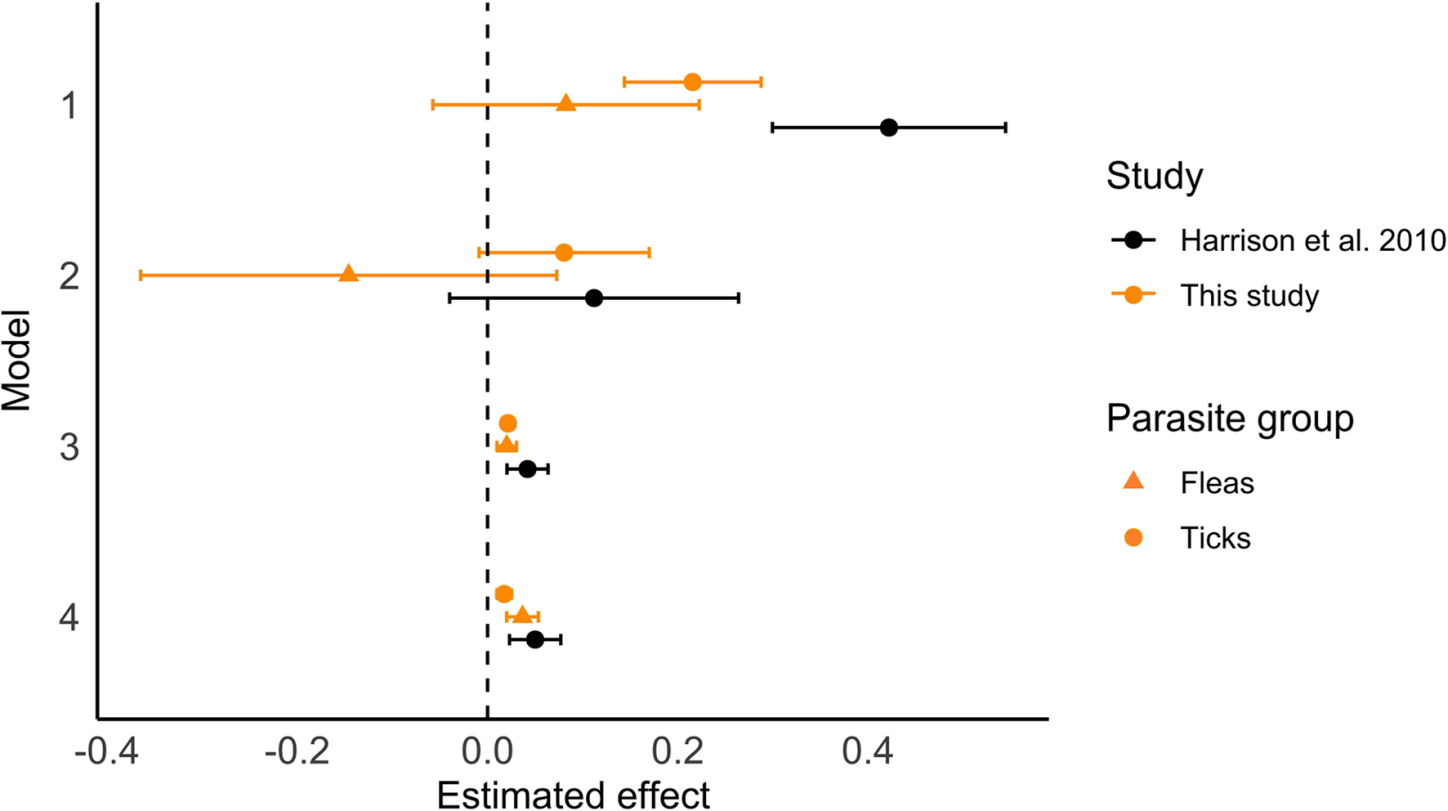
Estimated effect of sex (model 1 and 2) and body mass (models 3 and 4) of *A. flavicollis* (yellow, this study) and *A. sylvaticus* (black, Harrison et al. 2010) on their tick (circles) and flea (triangles) burdens. Error bars correspond to 95% confidence intervals. See Tables 1 and 2 for more information on the models

The study we here quasi-replicated (Harrison et al. 2010, Fig. 3) also had found male mice to carry more ticks and concluded that this pattern could be related to sexual size dimorphism. Our study, conducted on a different rodent species (*A. flavicollis* rather than *A. sylvaticus*), in a different geographic location (Poland vs. Ireland), in a different forest type (beech vs. mixed broadleaf and coniferous), and with a considerably larger sample size (1214 vs. 288 mice), produced similar findings. This convergence of results indicates that the relationship between *I. ricinus* and its hosts *Apodemus* spp. is robust. Our study showed a similarity in the pattern of sex bias in tick burdens in *Apodemus* spp. between Ireland and Poland when comparing the effect sizes of both studies (Fig. 3). However, the effect sizes observed in our study were consistently smaller than those in the original study. A comparison of effect sizes of both studies demonstrated that sex bias in tick burdens in *Apodemus* spp. followed a similar pattern in both Ireland and Poland. On the other hand, the effect sizes that we detected were consistently smaller than the ones from the original study. In particular, the effect of sex on tick loads, while significant, was weaker in our study.

In contrast to ticks, there was no evidence of male bias in flea parasitism (models 1 and 2). Similar results were reported by other authors (Benedek and Sirbu 2016; Kowalski et al. 2015). Interestingly, our models 3 and 4 demonstrated that that both males and females alike, heavier individuals carried higher loads of fleas (a pattern not detected in Kowalski et al. 2015, perhaps because of their smaller sample size). The most likely explanation for this pattern is that, even though flea abundance is affected by body size, and body size is influenced by sex, these effects were not strong enough to generate a clear-cut difference in flea infestation between males and females.

The positive effect of body mass on tick and flea infestation that we observed might be caused by several factors. Firstly, larger-bodied hosts could be easier targets to find and colonize (Hawlena et al. 2005; Harrison et al. 2010; Kiffner et al. 2013), which is especially relevant for parasites that actively seek their hosts, such as ticks. Secondly, if a larger resource patch can sustain more inhabitants, bigger hosts should have a higher parasitic burden (Presley and Willig 2008). Bigger host can also favor coexistence among parasites by reducing both intra- and inter-specific competition, providing a greater variety of accessible niches and better resource division (Kuriset al. 1980; Gregory et al. 1996; Morand and Poulin 1998; Kiffner et al. 2013). Finally, it could be more difficult for smaller hosts to tolerate a high ectoparasitic burden. This could lead to size-dependent differences in grooming, which would result in lower numbers of ectoparasites in small-bodied hosts (Hart et al. 1992; Hawlena et al. 2008). Self-grooming is a time-consuming activity that may be less critical for larger individuals, as they can access resources such as food and mates more easily and are often in better body condition, which allows them to compensate for the energy lost due to parasite infestation. Furthermore, the energy loss caused by parasites is relatively less significant for larger individuals than for smaller ones, making it more viable for larger hosts to neglect thorough cleaning of their fur. A larger body requires longer bouts of self-grooming to keep the parasite population at bay. Therefore, larger animals are either forced to spend proportionately more time on self-grooming or tolerate relatively higher parasite loads to engage in other vital activities such as foraging or reproduction (Raveh et al. 2011).

Furthermore, body mass is linked with other traits that may affect parasite acquisition. In several rodent species, male body mass has been demonstrated to have a positive correlation with home range size (Borowski 2003). Defending a larger home range requires increased mobility and social interactions, which can lead to a heightened risk of parasitism (Gregory et al. 1996; Jetz et al. 2004; Kiffner et al. 2014). In addition, higher testosterone levels in males are associated with greater body mass and testes size, causing behavioral changes that elevate the risk of parasite transmission through fights with competitors and mating (Forbes 1985; Royland et al. 1994; Breed and Taylor 2000).

In contrast, female mice tend to be less mobile and have smaller home ranges (Bergstedt 1966; Attuquayefio et al. 1986; Stradiotto et al. 2009). Additionally, female hormones such as estrogen are believed to have an immunostimulating effect, unlike testosterone (Klein 2004). On the other hand, females tend to have more social interactions than males, staying closer to the natal site after the juvenile stage and residing in nests with their offspring to provide parental care (Wolff 2007). Aggregation is considered a risk factor for parasitism, as it intensifies transmission rates (Anderson & May 1979; May and Anderson 1979; Arneberg et al. 1998; Krasnov et al. 2002; Christe et al. 2007).

Our findings highlight the importance of the sexual size dimorphism in shaping sex-biased parasitism patterns among small mammals (Moore and Wilson 2002; Harrison et al. 2010; Kowalski et al. 2015; Merabet et al. 2021, but see Morand et al. 2004; Krasnov et al. 2005a, b; Perez-Orella and Schulte-Hostedde 2005; Gorrell Jamieson and Schulte-Hostedde 2008). The mechanisms driving sex-biased parasitism can be intricate and involve interactions between various host and parasite traits, as well as environmental factors. Nevertheless, our study’s results are consistent with a similar study on a congeneric rodent species conducted in a different geographical location, which aids in generalizing tick parasitism patterns. The sex bias in flea infestations of *Apodemus* spp. appears to be less clear and might depend on the host species (Morand et al. 2004; Kiffner et al. 2013; Kowalski et al. 2015). While numerous studies have reported a link between ectoparasite infestation and host body size, the relationship is not consistently demonstrated and might vary across study systems and is not consistently demonstrated (Perez-Orella and Schulte-Hostedde 2005; Krasnov et al. 2011; 2012; Kiffner et al. 2014; Herrero-Cófreces et al. 2021). This varying relationship between ectoparasite infestation and host body size has broad implications for both host and parasite ecology and evolution, as well as epidemiological applications for control of zoonotic infections.

### Supplementary Information

Below is the link to the electronic supplementary material.

Supplementary file1 (PNG 90.7 kb)

High resolution image (EPS 153 KB)

Supplementary file2 (PNG 90.4 kb)

High resolution image (ESP 186 KB)

Supplementary file3 (DOCX 102 KB)

## Data Availability

The datasets used for analyses are available from the corresponding author upon request.
