# Correction to: Host body mass, not sex, affects ectoparasite loads in yellow‑necked mouse *Apodemus flavicollis*

**DOI:** 10.1007/s00436-023-08015-x

**Published:** 2023-11-16

**Authors:** Milena Zduniak, Sarah Serafini, Aleksandra Wróbel, Rafał Zwolak

**Affiliations:** 1 Department of Systematic Zoology, Adam Mickiewicz University in Poznań, Uniwersytetu Poznańskiego 6, 61‑614 Poznań, Poland; 2 Department of Zoology, Poznań University of Life Sciences, Wojska Polskiego 71C, 60‑625 Poznań, Poland

**Correction to: Parasitology Research (2023) 122:2599–2607**

**https://doi.org/10.1007/s00436-023-07958-5**

The authors regret that the version of Figure 3 that appears in the original article is incorrect. Accidentally in the final version of the published article the wrong version of figure 3 was uploaded and the confidence intervals don’t match the results from the models. The correct Figure 3 is shown below.
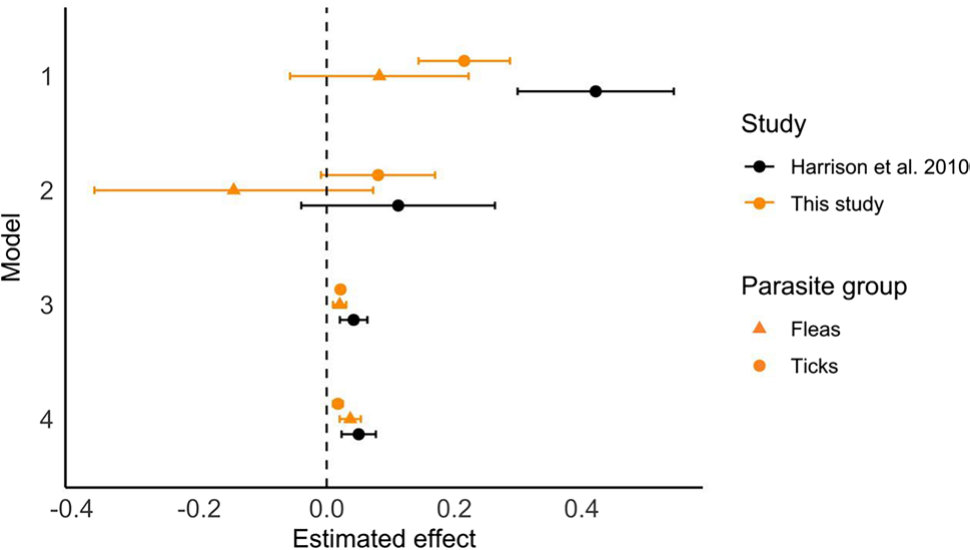


The original article has been corrected.

