# Supplementary figures and images for: Host body mass, not sex, affects ectoparasite loads in yellow-necked mouse Apodemus flavicollis
Source: Parasitol Res. 2023 Sep 13;122(11):2599–607. doi: 10.1007/s00436-023-07958-5 (PMC10567855; doi:10.1007/s00436-023-07958-5)

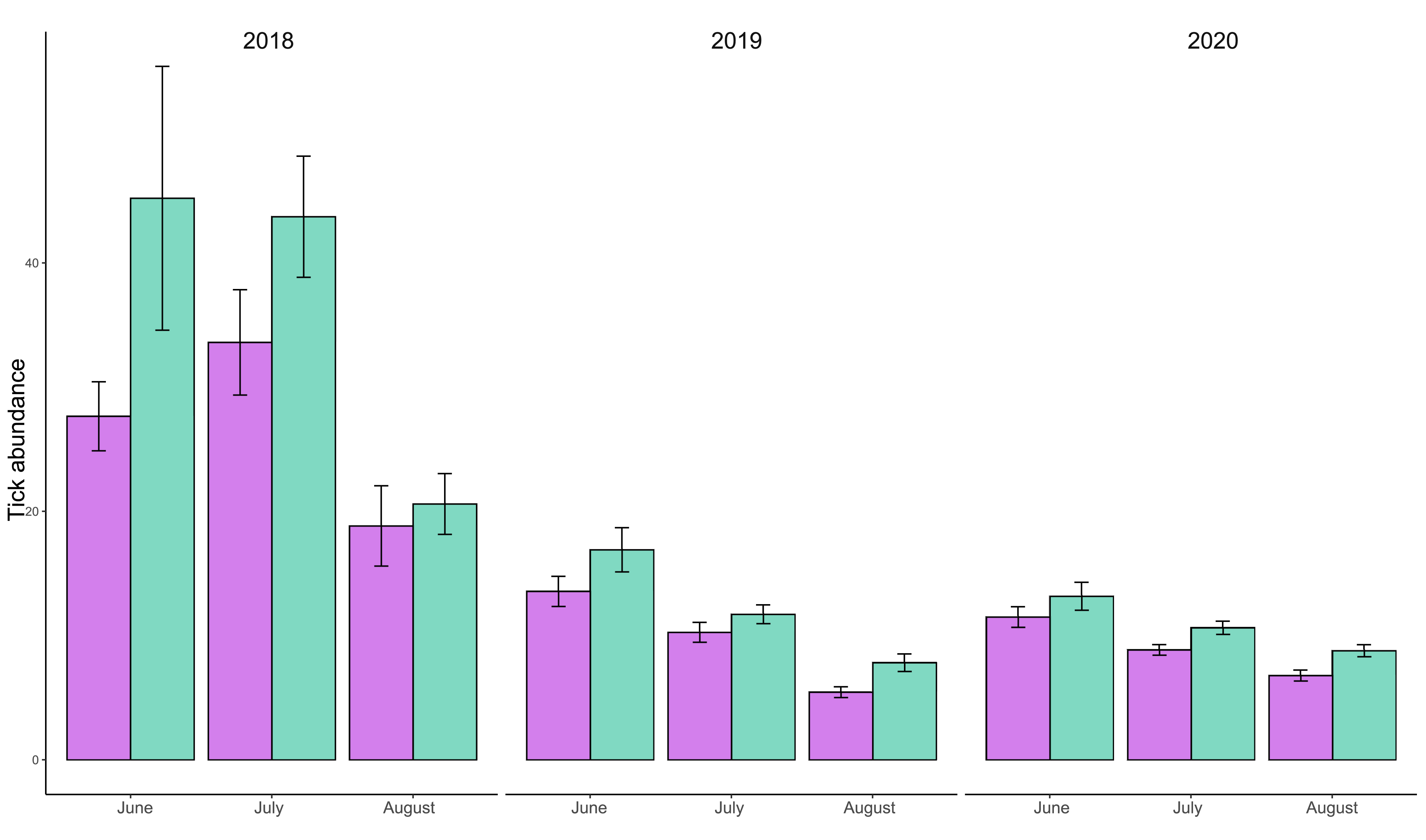

Supplement: Supplementary file 1 — Supplementary file1 (PNG 90.7 kb) [file 436_2023_7958_Fig4_ESM.png]

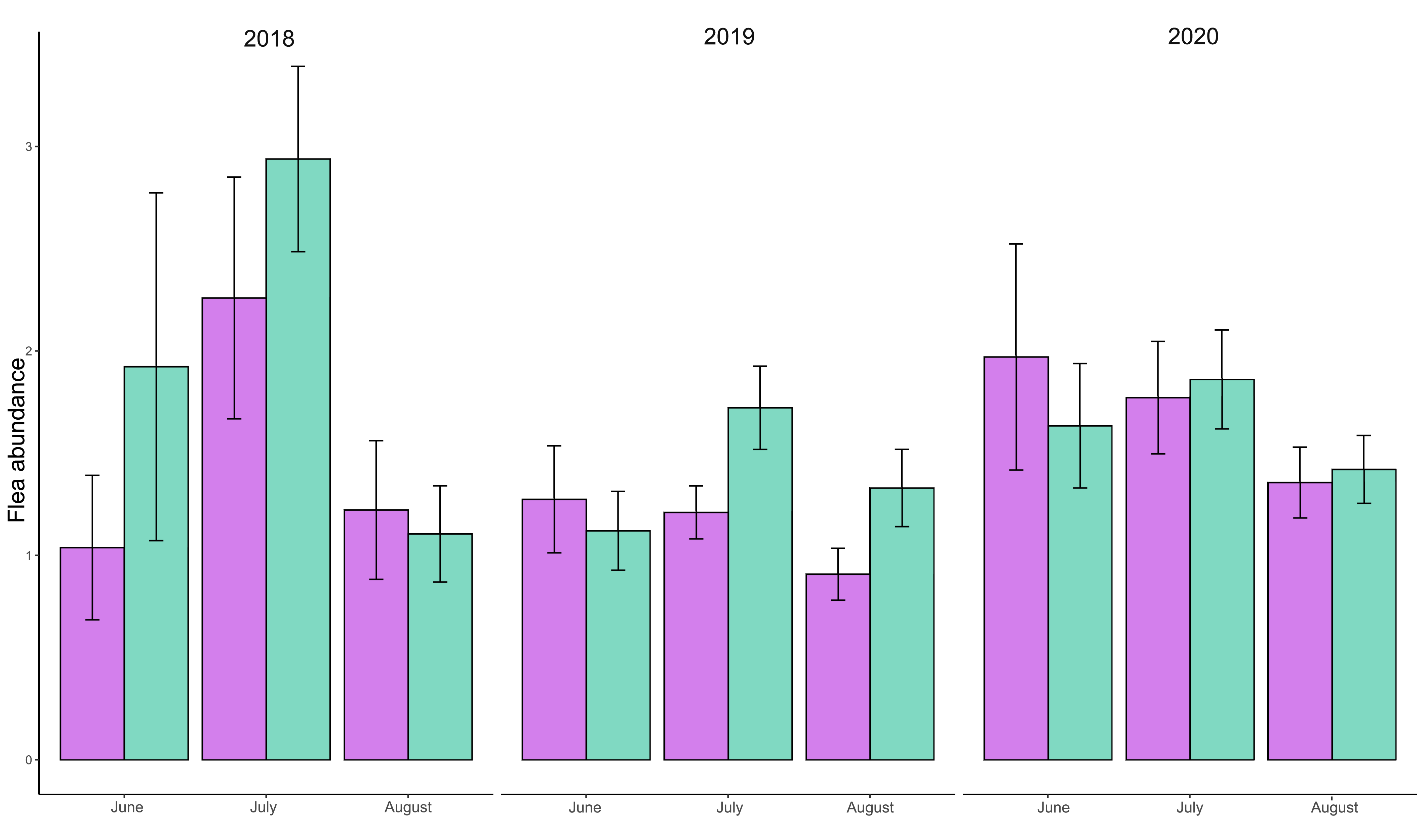

Supplement: Supplementary file 3 — Supplementary file2 (PNG 90.4 kb) [file 436_2023_7958_Fig5_ESM.png]
