# Supplementary material for: Host body mass, not sex, affects ectoparasite loads in yellow-necked mouse Apodemus flavicollis
Source: Parasitol Res. 2023 Sep 13;122(11):2599–607. doi: 10.1007/s00436-023-07958-5 (PMC10567855; doi:10.1007/s00436-023-07958-5)
Supplement: Supplementary file 5 — Supplementary file3 (DOCX 102 KB) [file 436_2023_7958_MOESM3_ESM.docx]

Table 1. Summary of models estimating the relationship between sex (model 1 and 2) and body mass (models 3 and 4) with tick loads in yellow-necked mice (Apodemus flavicollis). The Intercept corresponds to tick loads in female mice in August 2018. Sex M indicates the change in tick abundance for male hosts relative to females, Mass signifies the change in tick abundance per one gram increase in host body mass, Month June and Month July represent changes in tick abundance compared to August, and Year 2019 and Year 2020 denote changes in tick abundance relative to year 2018.

|  | *Model 1 (all)* | | | | *Model 2 (paired)* | | | | *Model 3 (only males)* | | | | | *Model 4 (only females)* | | | |
| --- | --- | --- | --- | --- | --- | --- | --- | --- | --- | --- | --- | --- | --- | --- | --- | --- | --- |
| *Predictors* | *β* | *SE* | *z* | *p* | *β* | *SE* | *z* | *p* | *β* | *SE* | *z* | *p* | *β* | | *SE* | *z* | *p* |
| (Intercept) | 3.00 | 0.08 | 36.01 | **<0.001** | 3.10 | 0.10 | 29.83 | **<0.001** | 2.58 | 0.14 | 18.96 | **<0.001** | 2.53 | | 0.16 | 15.38 | **<0.001** |
| Sex M | 0.22 | 0.04 | 4.95 | **<0.001** | 0.08 | 0.05 | 1.48 | 0.138 |  |  |  |  |  | |  |  |  |
| Mass |  |  |  |  |  |  |  |  | 0.02 | 0.00 | 6.30 | **<0.001** | 0.02 | | 0.00 | 3.76 | **<0.001** |
| Month July | 0.38 | 0.04 | 9.25 | **<0.001** | 0.38 | 0.07 | 5.56 | **<0.001** | 0.40 | 0.06 | 7.00 | **<0.001** | 0.48 | | 0.06 | 7.96 | **<0.001** |
| Month June | 0.64 | 0.06 | 11.07 | **<0.001** | 0.54 | 0.09 | 6.02 | **<0.001** | 0.70 | 0.08 | 8.90 | **<0.001** | 0.68 | | 0.08 | 8.33 | **<0.001** |
| Year 2019 | -1.20 | 0.06 | -19.22 | **<0.001** | -1.24 | 0.09 | -13.61 | **<0.001** | -1.26 | 0.08 | -15.45 | **<0.001** | -1.24 | | 0.09 | -13.52 | **<0.001** |
| Year 2020 | -1.14 | 0.07 | -16.88 | **<0.001** | -1.18 | 0.10 | -12.30 | **<0.001** | -1.18 | 0.09 | -13.85 | **<0.001** | -1.12 | | 0.10 | -11.58 | **<0.001** |
| Observations | 1224 | | | | 481 | | | | 663 | | | | | 561 | | | |

Table 2. Summary of models estimating the association between sex (model 1 and 2) and body mass (models 3 and 4) with flea loads in yellow-necked mice (Apodemus flavicollis). See Table 1 for details.

|  | *Model 1 (all)* | | | | *Model 2 (paired)* | | | | *Model 3 (only males)* | | | | *Model 4 (only females)* | | | |
| --- | --- | --- | --- | --- | --- | --- | --- | --- | --- | --- | --- | --- | --- | --- | --- | --- |
| *Predictors* | *β* | *SE* | *z* | *p* | *β* | *SE* | *z* | *p* | *β* | *SE* | *z* | *p* | *β* | *SE* | *Z* | *P* |
| (Intercept) | 0.39 | 0.15 | 2.63 | **0.008** | 0.63 | 0.23 | 2.76 | **0.006** | -0.11 | 0.25 | -0.46 | 0.643 | -0.61 | 0.36 | -1.70 | 0.088 |
| Sex M | 0.08 | 0.09 | 0.97 | 0.332 | -0.15 | 0.13 | -1.10 | 0.272 |  |  |  |  |  |  |  |  |
| Mass |  |  |  |  |  |  |  |  | 0.02 | 0.01 | 3.23 | **0.001** | 0.04 | 0.01 | 3.64 | **<0.001** |
| Month July | 0.37 | 0.09 | 4.13 | **<0.001** | 0.34 | 0.15 | 2.35 | **0.019** | 0.43 | 0.12 | 3.65 | **<0.001** | 0.40 | 0.14 | 2.92 | **0.004** |
| Month June | 0.28 | 0.13 | 2.20 | **0.028** | 0.25 | 0.21 | 1.16 | 0.245 | 0.27 | 0.17 | 1.63 | 0.102 | 0.36 | 0.19 | 1.87 | 0.062 |
| Year 2019 | -0.42 | 0.14 | -3.10 | **0.002** | -0.64 | 0.22 | -2.94 | **0.003** | -0.41 | 0.16 | -2.56 | **0.010** | -0.50 | 0.23 | -2.24 | **0.025** |
| Year 2020 | -0.16 | 0.14 | -1.19 | 0.235 | -0.34 | 0.21 | -1.61 | 0.107 | -0.26 | 0.15 | -1.68 | 0.093 | -0.09 | 0.22 | -0.40 | 0.692 |
| Observations | 1183 | | | | 469 | | | | 636 | | | | 547 | | | |

|  | *Model 1 (all)* | | | | *Model 2 (paired)* | | | | *Model 3 (only males)* | | | | *Model 4 (only females)* | | | | |
| --- | --- | --- | --- | --- | --- | --- | --- | --- | --- | --- | --- | --- | --- | --- | --- | --- | --- |
| *Juveniles included* | *β* | *SE* | *z* | *p* | *β* | *SE* | *z* | *p* | *β* | *SE* | *z* | *p* | *β* | *SE* | *z* | *p* |  |
|  | 0.21 | 0.04 | 5.03 | **<0.001** | 0.07 | 0.05 | 1.28 | 0.201 | 0.02 | 0 | 7.74 | **<0.001** | 0.02 | 0 | 4.44 | **<0.001** |  |
|  | N=1389 | | | | N=520 | | | | N=709 | | | | N=620 | | | | |
| *Juveniles < 14 g* | *β* | *SE* | *z* | *p* | *β* | *SE* | *z* | *p* | *β* | *SE* | *z* | *p* | *β* | *SE* | *z* | *p* |  |
|  | 0.22 | 0.04 | 4.95 | **<0.001** | 0.07 | 0.05 | 1.38 | 0.169 | 0.02 | 0 | 6.61 | **<0.001** | 0.02 | 0 | 3.75 | **<0.001** |  |
|  | N=1224 | | | | N=488 | | | | N=677 | | | | N=566 | | | | |
| *Juveniles < 15 g* | *β* | *SE* | *z* | *p* | *β* | *SE* | *z* | *p* | *β* | *SE* | *z* | *p* | *β* | *SE* | *z* | *p* |  |
|  | 0.22 | 0.04 | 4.95 | **<0.001** | 0.08 | 0.05 | 1.47 | 0.142 | 0.02 | 0 | 6.3 | **<0.001** | 0.02 | 0 | 3.76 | **<0.001** |  |
|  | N=1224 | | | | N=476 | | | | N=663 | | | | N=561 | | | | |
| *Juveniles < 16 g* | *β* | *SE* | *z* | *p* | *β* | *SE* | *z* | *p* | *β* | *SE* | *z* | *p* | *β* | *SE* | *z* | *p* |  |
|  | 0.21 | 0.04 | 4.85 | **<0.001** | 0.08 | 0.06 | 1.43 | 0.152 | 0.02 | 0 | 6.28 | **<0.001** | 0.02 | 0 | 3.68 | **<0.001** |  |
|  | N=1198 | | | | N=466 | | | | N=656 | | | | N=542 | | | | |
| *Juveniles < 17 g* | *β* | *SE* | *z* | *p* |  |  |  |  |  |  |  |  |  |  |  |  |  |
|  | 0.21 | 0.05 | 4.7 | **<0.001** | 0.07 | 0.06 | 1.31 | 0.189 | 0.02 | 0 | 6.5 | **<0.001** | 0.02 | 0.01 | 3.69 | **<0.001** |  |
|  | N=1155 | | | | N=452 | | | | N=644 | | | | N=511 | | | | |
| *Juveniles < 18 g* | *β* | *SE* | *z* | *p* | *β* | *SE* | *z* | *p* | *β* | *SE* | *z* | *p* | *β* | *SE* | *z* | *p* |  |
|  | 0.2 | 0.05 | 4.46 | **<0.001** | 0.07 | 0.06 | 1.28 | 0.201 | 0.02 | 0 | 6.62 | **<0.001** | 0.02 | 0.01 | 3.51 | **<0.001** |  |
|  | N=1117 | | | | N=442 | | | | N=631 | | | | N=486 | | | | |
| *Juveniles < 19 g* | *β* | *SE* | *z* | *p* | *β* | *SE* | *z* | *p* | *β* | *SE* | *z* | *p* | *β* | *SE* | *z* | *p* |  |
|  | 0.19 | 0.05 | 4.1 | **<0.001** | 0.07 | 0.06 | 1.23 | 0.219 | 0.02 | 0 | 6.65 | **<0.001** | 0.02 | 0.01 | 2.86 | **0.004** |  |
|  | N=1070 | | | | N=424 | | | | N=611 | | | | N=459 | | | | |
| *Juveniles < 20 g* | *β* | *SE* | *z* | *p* | *β* | *SE* | *z* | *p* | *β* | *SE* | *z* | *p* | *β* | *SE* | *z* | *p* |  |
|  | 0.19 | 0.05 | 3.91 | **<0.001** | 0.07 | 0.06 | 1.22 | 0.221 | 0.02 | 0 | 6.29 | **<0.001** | 0.02 | 0.01 | 2.47 | **0.014** |  |
|  | N=1013 | | | | N=397 | | | | N=592 | | | | N=421 | | | | |

Table 3. Summary of models estimating the association between sex (model 1 and 2) and body mass (models 3 and 4) with tick loads of yellow-necked mouse Apodemus flavicollis including juveniles or excluding them at different body mass thresholds.

Table 4. Summary of models estimating the association between sex (model 1 and 2) and body mass (models 3 and 4) with flea loads of yellow-necked mouse Apodemus flavicollis including juveniles or excluding them at different body mass thresholds.

|  | *Model 1 (all)* | | | | *Model 2 (paired)* | | | | *Model 3 (only males)* | | | | *Model 4 (only females)* | | | | |
| --- | --- | --- | --- | --- | --- | --- | --- | --- | --- | --- | --- | --- | --- | --- | --- | --- | --- |
| *Juveniles included* | *β* | *SE* | *z* | *p* | *β* | *SE* | *z* | *p* | *β* | *SE* | *z* | *p* | *β* | *SE* | *z* | *p* |  |
|  | 0.14 | 0.08 | 1.75 | 0.08 | -0.11 | 0.13 | -0.83 | 0.406 | 0.21 | 0.05 | 3.92 | **<0.001** | 0.04 | 0.01 | 4.45 | **<0.001** |  |
|  | N=1346 | | | | N=513 | | | | N=679 | | | | N=602 | | | | |
| *Juveniles < 14 g* | *β* | *SE* | *z* | *p* | *β* | *SE* | *z* | *p* | *β* | *SE* | *z* | *p* | *β* | *SE* | *z* | *p* |  |
|  | 0.08 | 0.08 | 0.92 | 0.356 | -0.15 | 0.13 | -1.12 | 0.261 | 0.17 | 0.05 | 3.24 | **0.001** | 0.04 | 0.01 | 3.53 | **<0.001** |  |
|  | N=1199 | | | | N=474 | | | | N=648 | | | | N=551 | | | | |
| *Juveniles < 15 g* | *β* | *SE* | *z* | *p* | *β* | *SE* | *z* | *p* | *β* | *SE* | *z* | *p* | *β* | *SE* | *z* | *p* |  |
|  | 0.08 | 0.09 | 0.97 | 0.332 | -0.17 | 0.13 | -1.31 | 0.19 | 0.17 | 0.05 | 3.23 | **0.001** | 0.04 | 0.01 | 3.64 | **<0.001** |  |
|  | N=1183 | | | | N=462 | | | | N=636 | | | | N=547 | | | | |
| *Juveniles < 16 g* | *β* | *SE* | *z* | *p* | *β* | *SE* | *z* | *p* | *β* | *SE* | *z* | *p* | *β* | *SE* | *z* | *p* |  |
|  | 0.07 | 0.09 | 0.83 | 0.407 | -0.19 | 0.13 | -1.46 | 0.143 | 0.18 | 0.05 | 3.38 | **0.001** | 0.04 | 0.01 | 3.46 | **0.001** |  |
|  | N=1157 | | | | N=454 | | | | N=629 | | | | N=528 | | | | |
| *Juveniles < 17 g* | *β* | *SE* | *z* | *p* | *β* | *SE* | *z* | *p* | *β* | *SE* | *z* | *p* | *β* | *SE* | *z* | *p* |  |
|  | 0.04 | 0.09 | 0.5 | 0.617 | -0.19 | 0.13 | -1.45 | 0.148 | 0.18 | 0.05 | 3.3 | **0.001** | 0.03 | 0.01 | 2.81 | **0.005** |  |
|  | N=1117 | | | | N=441 | | | | N=617 | | | | N=500 | | | | |
| *Juveniles < 18 g* | *β* | *SE* | *z* | *p* | *β* | *SE* | *z* | *p* | *β* | *SE* | *z* | *p* | *β* | *SE* | *z* | *p* |  |
|  | 0.01 | 0.09 | 0.13 | 0.897 | -0.22 | 0.13 | -1.66 | 0.096 | 0.2 | 0.06 | 3.49 | **<0.001** | 0.03 | 0.01 | 2.26 | **0.024** |  |
|  | N=1080 | | | | N=431 | | | | N=605 | | | | N=475 | | | | |
| *Juveniles < 19 g* | *β* | *SE* | *z* | *p* | *β* | *SE* | *z* | *p* | *β* | *SE* | *z* | *p* | *β* | *SE* | *z* | *p* |  |
|  | 0.03 | 0.09 | 0.32 | 0.751 | -0.21 | 0.14 | -1.54 | 0.123 | 0.19 | 0.06 | 3.33 | **0.001** | 0.03 | 0.01 | 2.48 | **0.013** |  |
|  | N=1034 | | | | N=405 | | | | N=584 | | | | N=450 | | | | |
| *Juveniles < 20 g* | *β* | *SE* | *z* | *p* | *β* | *SE* | *z* | *p* | *β* | *SE* | *z* | *p* | *β* | *SE* | *z* | *p* |  |
|  | 0.02 | 0.09 | 0.18 | 0.858 | -0.17 | 0.14 | -1.21 | 0.227 | 0.16 | 0.06 | 2.78 | **0.005** | 0.02 | 0.01 | 1.84 | 0.066 |  |
|  | N=981 | | | | N=383 | | | | N=564 | | | | N=417 | | | | |


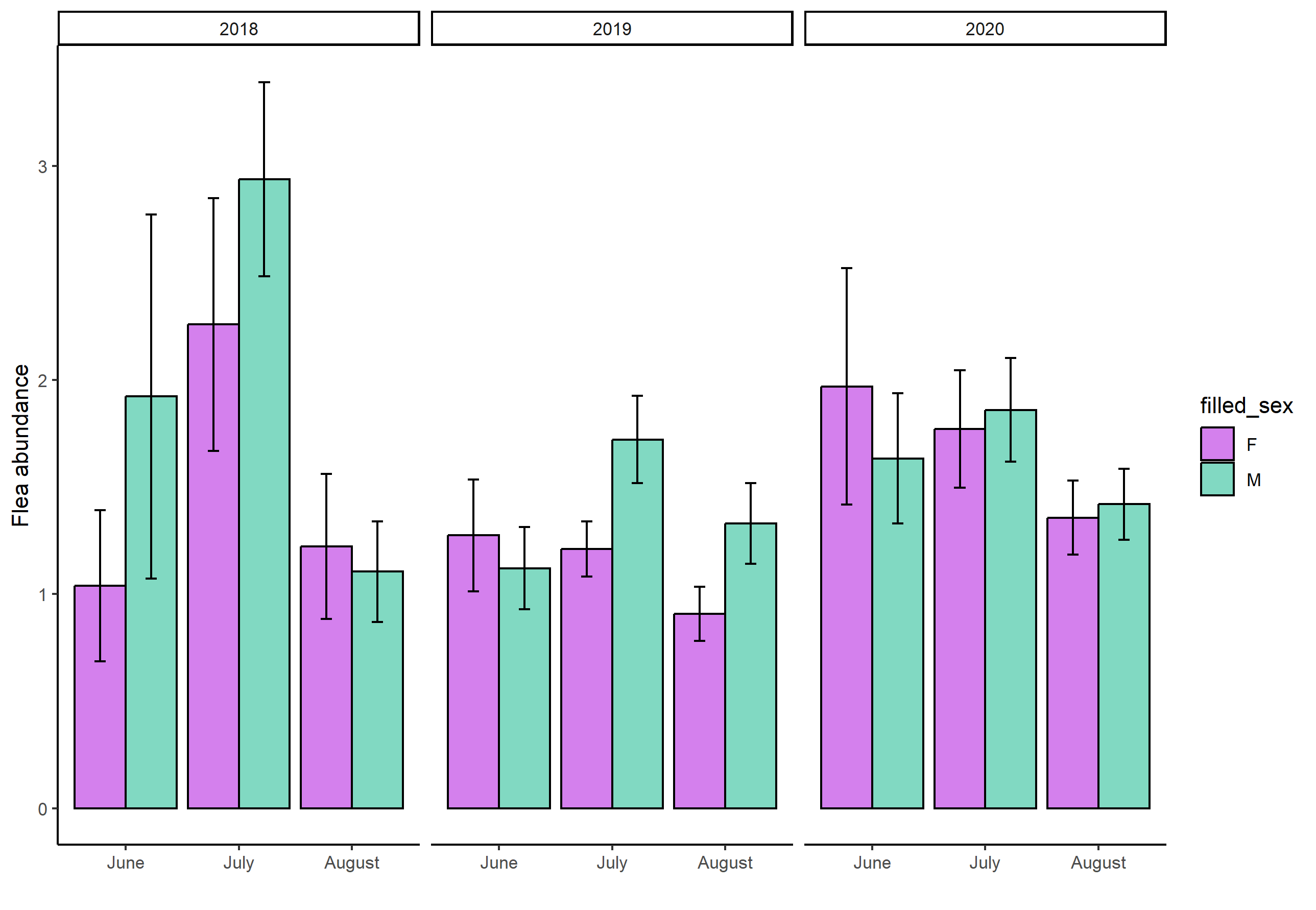

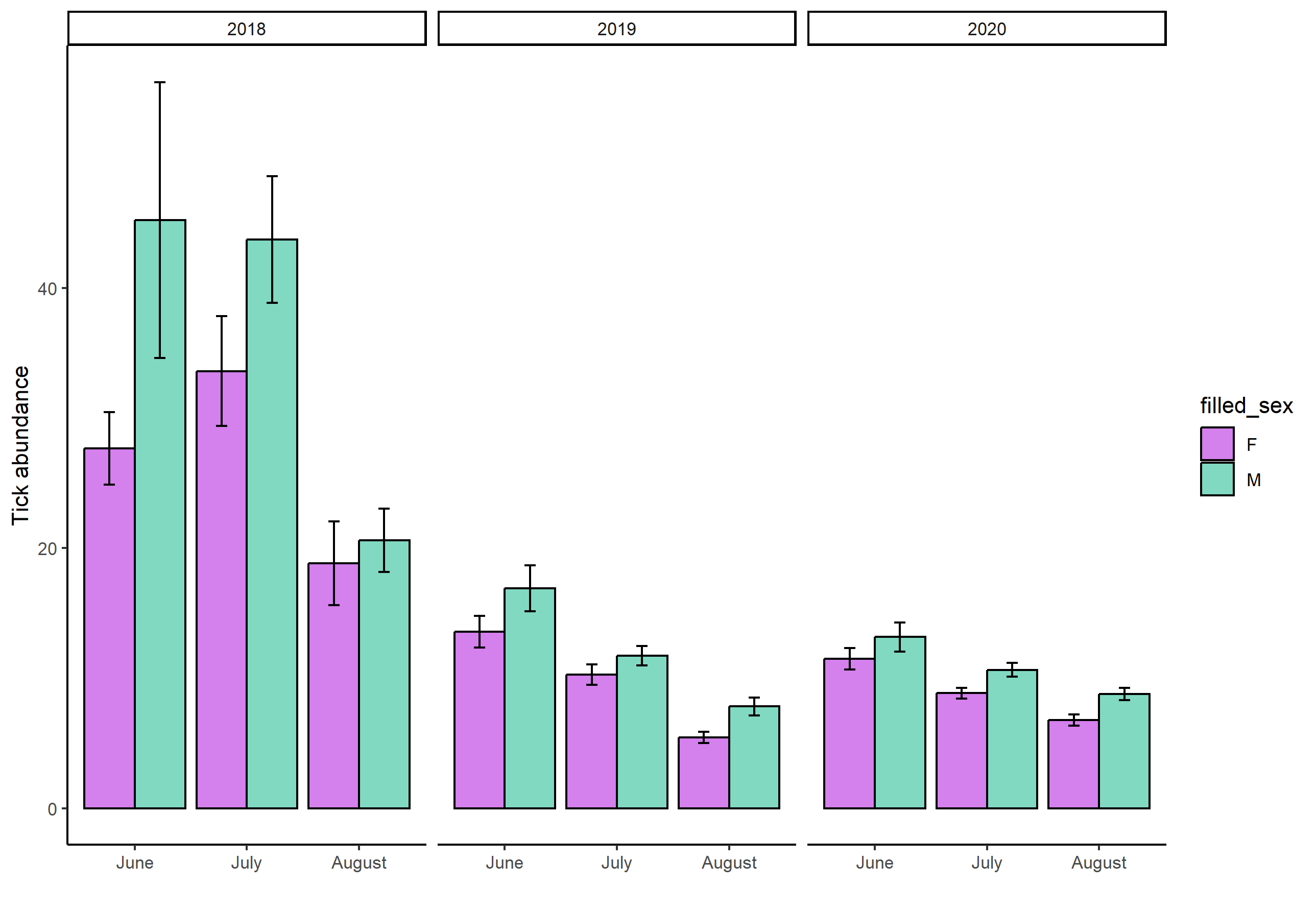


Figure 2. Yearly and monthly average (+- SE) abundance of fleas on female ( violet) and male (green) yellow-necked mouse Apodemus flavicollis.

Figure 1. Yearly and monthly average (+- SE) abundance of tick Ixodes ricinus on female ( violet) and male (green) yellow-necked mouse Apodemus flavicollis.
